# Predictors of Mortality in Critically Ill Patients With Antineutrophil Cytoplasmic Antibody-Associated Vasculitis

**DOI:** 10.3389/fmed.2021.762004

**Published:** 2021-10-25

**Authors:** Yuqi Zhang, Jinyan Guo, Panpan Zhang, Lei Zhang, Xiaoguang Duan, Xiaofei Shi, Nailiang Guo, Shengyun Liu

**Affiliations:** ^1^Department of Rheumatology and Immunology, the First Affiliated Hospital of Zhengzhou University, Zhengzhou, China; ^2^Department of Intensive Care Unit, the First Affiliated Hospital of Zhengzhou University, Zhengzhou, China; ^3^Department of Rheumatology, the First Affiliated Hospital and College of Clinical Medicine, Henan University of Science and Technology, Luoyang, China; ^4^Department of Rheumatology and Immunology, Xinyang Central Hospital, Xinyang, China

**Keywords:** antineutrophil cytoplasmic antibody-associated vasculitis, intensive care unit, mortality, predictors, Acute Physiology and Chronic Health Evaluation II, nomogram

## Abstract

**Background:** Patients with antineutrophil cytoplasmic antibody-associated vasculitis (AAV) may require intensive care unit (ICU) admission due to different reasons, and the in-ICU mortality is high among AAV patients. The aim of this study was to explore the clinical features and risk factors of mortality of patients with AAV in the ICU.

**Methods:** A retrospective study was conducted based on 83 AAV patients admitted to the ICU in a tertiary medical institution in China. Data on clinical characteristics, laboratory tests, treatment in ICU and outcomes were collected. The data were analyzed using univariate and multivariate logistic regression analysis to explore the variables that were independently related to mortality. Kaplan–Meier method was used to assess the long-term survival.

**Results:** Among the 83 patients, 41 (49.4%) were female. The mean age of patients was 66 ± 13 years. Forty-four patients deceased, with the in-ICU mortality of 53%. The most common cause for ICU admission was active vasculitis (40/83, 48.2%). The main cause of death was infection (27/44, 61.4%) followed by active vasculitis (15/44, 34.1%). A multivariate analysis revealed that the Acute Physiology and Chronic Health Evaluation II (APACHE II) at ICU admission (*OR* = 1.333, 95% *CI*: 1.031–1.722) and respiratory failure (*OR* = 620.452, 95% *CI*: 11.495–33490.306) were independent risk factors of in-ICU death. However, hemoglobin (*OR* = 0.919, 95% *CI*: 0.849–0.995) was an independent protective factor. The nomogram established in this study was practical in predicting the risk of in-ICU mortality for AAV patients. Moreover, for 39 patients survived to the ICU stay, the cumulative survival rates at 0.5, 1, and 5 years were 58.3%, 54.2%, and 33.9%, respectively, and the median survival time was 14 months.

**Conclusion:** In our study, active vasculitis was the most frequent reason for ICU admission, and the main cause of death was infection. APACHE II and respiratory failure were independent risk factors while hemoglobin was an independent protective factor of in-ICU mortality for AAV patients admitted to the ICU. The risk prediction model developed in this study may be a useful tool for clinicians in early recognition of high-risk patients and applying appropriate management.

## Introduction

Antineutrophil cytoplasmic antibody (ANCA)-associated vasculitis (AAV) is a systemic necrotizing vasculitis that predominantly affects small vessels and is associated with the presence of ANCAs in serum (1, 2). AAV comprises three entities, including microscopic polyangiitis (MPA), granulomatosis with polyangiitis (GPA) and eosinophilic GPA (EGPA) (3). Early diagnosis of AAV combined with administration of immunotherapies such as glucocorticoids, immunosuppressive drugs and rituximab have significantly improved the survival rate of AAV patients (4, 5). However, some AAV patients may require intensive care unit (ICU) admission due to life-threatening manifestations at the time of diagnosis, disease flare-up or severe complications due to immunosuppressive therapies (6–8). AAV is one of the most frequent systemic rheumatic diseases (SRD) in the ICU (9, 10) with high mortality ranging from 15.5% to 58.8% (8, 11, 12). According to researches, the in-ICU mortality is higher among AAV patients compared with other SRD patients (13).

The number of studies on the clinical features and ICU outcome of AAV patients is limited. Previous studies have reported that infection and life-threatening manifestations of active vasculitis are the main reasons for ICU admission of AAV patients (8, 11). The prediction of patients' outcome is clinically important, especially for patients in the ICU. Different methods for measuring disease severity in ICU patients have been developed, including Acute Physiology and Chronic Health Evaluation II (APACHE II) (14), Sequential Organ Failure Assessment (SOFA) (15) and Simplified Acute Physiologic Score II (SAPS II) (16). These disease severity scores are computed at the time of admission to ICU and have been reported to be associated with ICU outcome of AAV patients in some studies (8, 11). However, the value of specific vasculitis score like Birmingham vasculitis activity score system (BVAS) on patients' outcome in the ICU is yet to be shown (6, 17). To date, few studies have investigated the relationship between clinical features and risk of mortality in the ICU for AAV patients. Most of the studies are small-sized and lack data about Asians. Therefore, further research in this area is needed.

To identify the predictors of in-ICU mortality for AAV patients in the Chinese population, we conducted a single-center, retrospective study. In the study, data about clinical characteristics, laboratory tests and treatment in ICU were collected. Our results revealed that active vasculitis was the most common reason for ICU admission and a novel model combined respiratory failure, hemoglobin and APACHE II is a good predictor of in-ICU mortality for AAV patients.

## Materials and Methods

### Patients and Study Design

**Figure 1:**
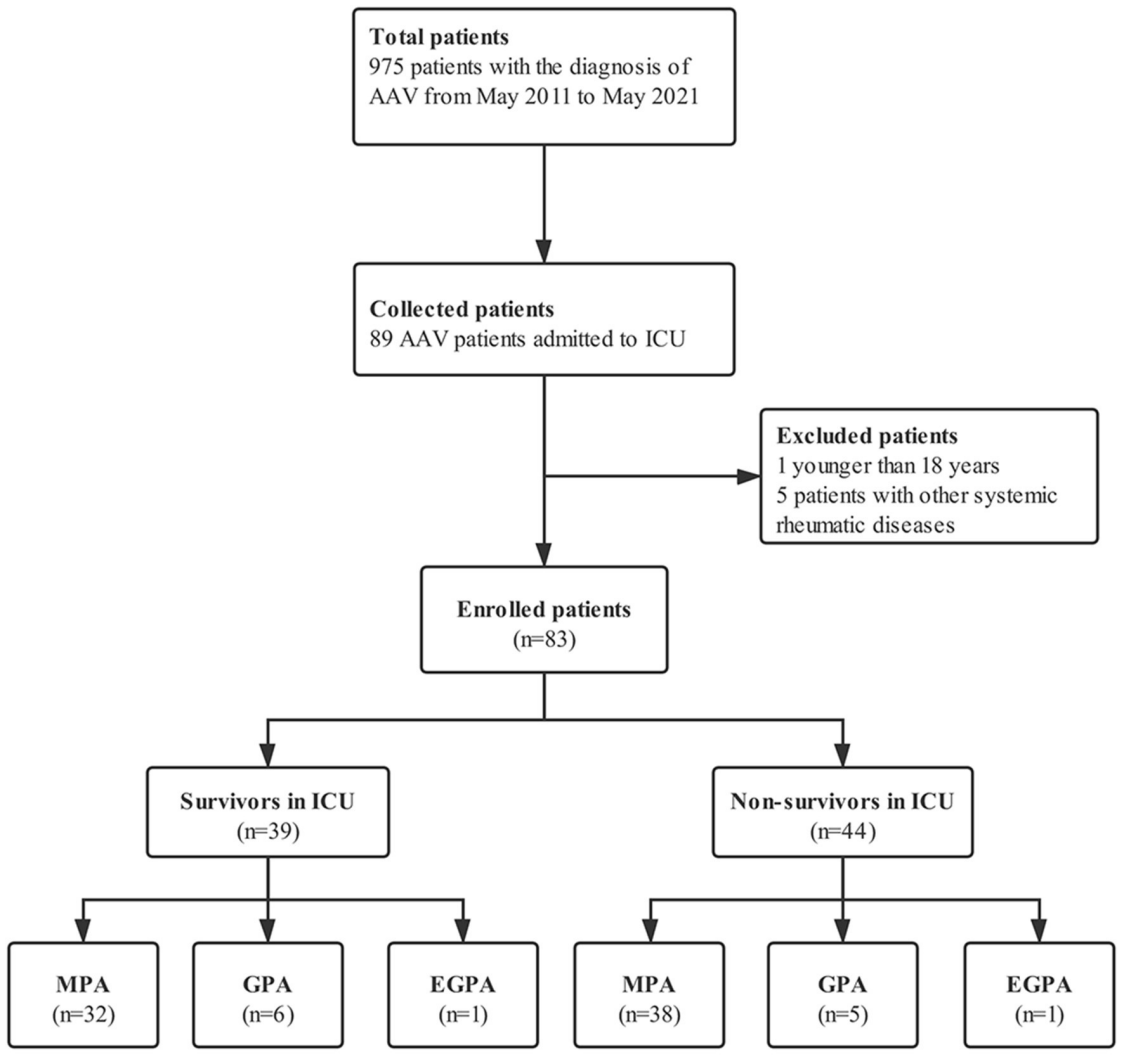
Flow chart of the study design.

In this retrospective study, patients with AAV admitted to the ICU of the First Affiliated Hospital of Zhengzhou University from May 2011 to May 2021 were identified. All patients fulfilled the 2012 revised International Chapel Hill Consensus Conference definitions for the AAV (3) or the American College of Rheumatology criteria for Wegener's granulomatosis and Churg-Strauss syndrome (18, 19). Exclusion criteria included patients with drug-induced AAV, patients who were less than 18 years or those with other SRDs such as systemic lupus erythematosus, rheumatoid arthritis, inflammatory myopathy and antiphospholipid syndrome. Consequently, among 975 patients diagnosed with AAV, we selected 83 patients who were admitted to the ICU during the study period (Figure 1). For patients admitted to the ICU more than once, only the first admission was considered. In this study, the primary outcome for AAV patients hospitalized in the ICU was in-ICU death. Patients who were discharged from ICU against medical advice and ended up deceased within 1 week were also considered to have undergone in-ICU death. Besides, long-term outcomes were also recorded of the patients survived to the first ICU stay. Our study complied with the Declaration of Helsinki and was approved by the Ethical Committee of the First Affiliated Hospital of Zhengzhou University (**No. 2021-KY-0610**).

### Clinical Data and Laboratory Examinations

In this study, we collected patients' data, including age, gender, medical history of chronic comorbidities (20), classification of AAV, reasons for ICU admission, length of stay in ICU, clinical features and organ involvement, treatment at the moment of ICU admission and during ICU hospitalization as well as patients' outcome. To assess AAV disease activity and severity, APACHE II (14) and BVAS (21) were calculated within the first 24 h of ICU admission. APACHE II is determined by total points from three sections, including age, chronic health status, and 12 acute physiologic variables. It is computed using the most deranged physiologic variables during the patient's initial 24 h in ICU, with a total score between 0 and 71 points (14). BVAS is computed by points from 10 systems (one general, eight tissue-specific and one open), with a maximum score for each system (21). Different laboratory tests were performed for ICU patients including routine blood analysis, liver function test, kidney function test, ANCA, arterial blood gas analysis and inflammatory indexes like C-reactive protein (CRP), erythrocyte sedimentation rate (ESR) and procalcitonin analysis.

### Definition of Terms

The causes for admission to the ICU were classified as active vasculitis, infection and other causes not attributed to infection or active vasculitis. Active vasculitis was diagnosed when new, persistent, or worsening clinical symptoms attributed to AAV and not related to prior organ damage were detected (22). Infection was defined by suspected clinical signs of infection accompanied with sufficient laboratory and imaging findings or microbiological results. Respiratory failure was diagnosed if oxygenation index (PaO_2_/FiO_2_) was <300 mmHg (23) or patient was in need of mechanical ventilation. Diffuse alveolar hemorrhage (DAH) was defined as the presence of hemoptysis, rapid decrease in hemoglobin and alveolar infiltrates on chest imaging or hemorrhagic bronchoalveolar lavage fluid accompanied with hemosiderin-laden macrophages (24). Interstitial lung disease (ILD) was defined according to British Thoracic Society guidelines, excluding the causes of infection, medication and pulmonary edema (25). Pulmonary arterial hypertension was diagnosed if the tricuspid regurgitation jet velocity was more than 2.8 m/s or systolic pulmonary artery pressure was more than 35 mmHg determined by echocardiography or confirmed by right heart catheterization (26, 27). Renal insufficiency was diagnosed by the presence of glomerular filtration rate (GFR) <60 ml/min per 1.73 m^2^, rapidly rising plasma creatinine or oliguria (urine volume <30 mL/h or 400 mL/day) (28). Glomerulonephritis was defined if proteinuria was >0.5 grams per 24 h accompanied with hematuria or confirmed renal biopsy. End-stage renal disease (ESRD), was defined as GFR <15 ml/min per 1.73 m^2^, in need of regular course of long-term renal-replacement therapy or kidney transplantation (28, 29). Cardiovascular diseases included acute myocardial infarction, arrhythmia, and the class IV cardiac function according to the New York Heart Association functional classification system (30). Shock was defined as systolic blood pressure <90 mmHg and/or diastolic blood pressure <60 mmHg with signs of organ hypoperfusion (31). Other manifestations of active vasculitis were defined according to BVAS (21).

### Statistical Analysis

Data were reported as means with standard deviation or median with inter-quartile range (Q1–Q3) for continuous variables and as frequencies or percentages for categorical data. Differences between survivors and non-survivors were explored using independent-samples *t*-tests or Mann–Whitney *U* test for continuous variables and Chi-square test or Fisher's exact test for categorical data. Univariate and multivariate logistic regression analysis was performed to explore the variables that were independently related to mortality. Laboratory tests, disease assessment scores and clinical features were treated as independent variables. In-ICU death was used as dependent variable. A nomogram was constructed based on the results of multivariate logistic regression analysis to predict the risk of in-ICU death. Besides, calibration curves, decision curve analysis (DCA) and receiver operating characteristic (ROC) curves were plotted to determine the reliability of our nomogram. Kaplan–Meier method was used to assess the long-term survival. A two-tailed *P*-value < 0.05 was considered statistically significant. Statistical analyses were performed using IBM SPSS Statistics software (Version 25.0), GraphPad Prism (Version 8.0.2) and R software (Version 3.6.1).

## Results

### Patients' Characteristics and Disease Severity Scoring

The characteristics of the patients and disease severity data are detailed in Table 1. Among the 83 patients admitted to the ICU, 41 (49.4%) were female and the mean age of all the patients was 66 ± 13 years. The type of AAV was established for all 83 patients, with MPA in 70 patients (84.3%), GPA in 11 patients (13.3%) and EGPA in 2 patients (2.4%). The groups between non-survivors and survivors were similar regarding gender, age, medical history and classification of AAV. Forty-eight patients (57.8%) were newly diagnosed with AAV. The median duration between AAV diagnosis and ICU admission in the remaining 35 patients was 12 (3–44) months. Thirty-nine patients (47%) were receiving glucocorticoids while therapies showed no statistical difference between groups at the moment of ICU admission. Disease severity assessed by APACHE II score at ICU admission was significantly higher in non-survivors' group [20.00 (15.00–25.75)] than that in survivors' group [12.00 (9.00–14.00)]. Disease activity assessed by BVAS score at ICU admission was also higher for non-survivors (25.16 ± 8.69) than that for survivors (15.67 ± 9.44).

**Table 1:** Demographic and clinical data of 83 AAV patients admitted to ICU.

**Parameters**	**Total** **(***n*** = 83)**	**Survivors** **(***n*** = 39)**	**Non-survivors** **(***n*** = 44)**	***P*** **-value**
Age (years, mean ± SD)	66 ± 13	65 ± 12	66 ± 13	0.886
Female gender (*n*, %)	41 (49.4)	20 (51.3)	21 (47.7)	0.746
Medical history (*n*, %)				
Diabetes	13 (15.7)	8 (20.5)	5 (11.4)	0.252
Hypertension	33 (39.8)	17 (43.6)	16 (36.4)	0.502
Coronary heart disease	14 (17.3)	6 (15.4)	8 (18.2)	0.734
Cerebrovascular disease	16 (19.3)	7 (17.9)	9 (20.5)	0.773
Malignancy	2 (2.4)	1 (2.6)	1 (2.3)	1.000
Classification of AAV (*n*, %)				0.859
MPA	70 (84.3)	32 (82.1)	38 (86.4)	
GPA	11 (13.3)	6 (15.4)	5 (11.4)	
EGPA	2 (2.4)	1 (2.6)	1 (2.3)	
Newly diagnosed as AAV (*n*, %)	48 (57.8)	22 (56.4)	26 (59.1)	0.805
Course of AAV (month), M (Q1–Q3)	12.00 (3.00–44.00)	10.50 (2.00–37.75)	18.00 (3.50–51.00)	0.366
Treatment at the moment of ICU admission				
Glucocorticoids	39 (47.0)	16 (41.0)	23 (52.3)	0.306
Cyclophosphamide	7 (8.4)	1 (2.6)	6 (13.6)	0.157
Mycophenolate mofetil	3 (3.6)	1 (2.6)	2 (4.5)	1.000
Azathioprine	1 (1.2)	0 (0)	1 (2.3)	1.000
Plasma exchange	2 (2.4)	1 (2.6)	1 (2.3)	1.000
Hemodialysis	12 (14.5)	6 (15.4)	6 (13.6)	0.821
Length of stay in ICU (day), M (Q1–Q3)	5 (2–10)	5 (2–11)	5 (3–8)	0.953
Disease and severity assessment scores				
APACHE II, M (Q1–Q3)	15.00 (11.00–21.00)	12.00 (9.00–14.00)	20.00 (15.00–25.75)	**<0.001**
BVAS (mean ± SD)	20.70 ± 10.18	15.67 ± 9.44	25.16 ± 8.69	**<0.001**

*Values highlighted in bold represent statistically significant P values (P < 0.05)*.

*AAV, antineutrophil cytoplasmic antibody-associated vasculitis; ICU, intensive care unit; MPA, microscopic polyangiitis; GPA, granulomatosis with polyangiitis; EGPA, eosinophilic granulomatosis with polyangiitis; APACHE II, Acute Physiology and Chronic Health Evaluation II; BVAS, Birmingham vasculitis active scoring system*.

### Causes of ICU Admission for AAV Patients

The causes of admission to the ICU for the 83 patients are displayed in Table 2. The most common reason for ICU admission was active vasculitis, accounting for 48.2% of the AAV patients. Active vasculitis cases included 17 with acute renal insufficiency, eight with diffuse alveolar hemorrhage, nine with pulmonary-renal syndrome, five with stroke and one with subglottic stenosis. For the 35 patients (42.2%) admitted to the ICU mainly due to infection, the most common cause was pneumonia. In addition, eight patients (9.6%) were admitted for other reasons, including pericardial tamponade, acute heart failure, mediastinal emphysema, etc.

**Table 2:** Causes of ICU admission of 83 AAV patients.

**Causes of ICU admission**	**Number of patients**
Active vasculitis (*n*, %)	40 (48.2)
Pulmonary-renal syndrome	9
Diffuse alveolar hemorrhage	8
Acute renal insufficiency	17
Stroke	5
Subglottic stenosis	1
Infection (*n*, %)	35 (42.2)
Pneumonia	29
Sepsis	5
Urinary tract infection	1
Other reasons (*n*, %)	8 (9.6)
Cancer cachexia	1
Pericardial tamponade	1
Acute heart failure	1
Exacerbation of dilated cardiomyopathy	1
Mediastinal emphysema	1
AE-COPD	1
Coma after cardiopulmonary resuscitation	1
Postoperative complication of anterior	1
chamber paracentesis	

*AAV, antineutrophil cytoplasmic antibody-associated vasculitis; ICU, intensive care unit; AE-COPD, acute exacerbation of chronic obstructive pulmonary disease*.

### Clinical Features and Laboratory Tests of Non-survivors and Survivors

Data for clinical features of the 83 patients in the ICU are provided in Table 3. Nineteen patients (22.9%) had DAH while 25 patients (30.1%) had ILD, though no significant difference was showed between non-survivors and survivors. Notably more non-survivors suffered from respiratory failure (95.5% vs. 23.1%) and pulmonary arterial hypertension (27.3% vs. 2.6%) than survivors. We further analyzed pulmonary involvement between cytoplasmic or proteinase-3-ANCA (c/PR3-ANCA)-positive and perinuclear or myeloperoxidase-ANCA (p/MPO-ANCA)-positive patients (Supplementary Table 1). In the total of 83 patients, 73 (88.0%) had a positive ANCA during the ICU admission. DAH was more frequent in c/PR3-ANCA-positive patients (50.0%) than p/MPO-ANCA-positive patients (22.2%). However, ILD was more common in p/MPO-ANCA-positive patients (31.7%) than c/PR3-ANCA-positive patients (10.0%), though no statistical significances were shown between the two groups regarding DAH and ILD. Besides, the groups of c/PR3-ANCA-positive and p/MPO-ANCA-positive patients were similar in terms of pulmonary arterial hypertension, pulmonary nodules, pulmonary infiltrates, pleural effusions, pulmonary embolism and respiratory failure.

**Table 3:** Clinical features of 83 AAV patients in ICU.

**Characteristics (n, %)**	**Total** **(***n*** = 83)**	**Survivors** **(***n*** = 39)**	**Non-survivors** **(***n*** = 44)**	***P*** **-value**
**Fever**	48 (57.8)	20 (51.3)	28 (63.6)	0.255
**Respiratory system**				
Cough	31 (37.3)	14 (35.9)	17 (38.6)	0.797
Hemoptysis	11 (13.3)	4 (10.3)	7 (15.9)	0.448
Diffuse alveolar hemorrhage	19 (22.9)	7 (17.9)	12 (27.3)	0.313
Interstitial lung disease	25 (30.1)	10 (25.6)	15 (34.1)	0.402
Pleural effusions	48 (57.8)	22 (56.4)	26 (59.1)	0.805
Pulmonary embolism	3 (3.6)	1 (2.6)	2 (4.5)	1.000
Pulmonary arterial hypertension	13 (15.7)	1 (2.6)	12 (27.3)	**0.002**
Respiratory failure	51 (61.4)	9 (23.1)	42 (95.5)	**<0.001**
**Renal disease**				
Renal insufficiency	59 (71.1)	24 (61.5)	35 (79.5)	0.071
Glomerulonephritis	38 (45.8)	14 (35.9)	24 (54.5)	0.089
**Cardiovascular disease**				
Heart failure	53 (63.9)	22 (56.4)	31 (70.5)	0.184
Acute myocardial infarction	5 (6.0)	1 (2.6)	4 (9.1)	0.432
Arrhythmia	3 (3.6)	1 (2.6)	2 (4.5)	1.000
**Digestive system**				
Gastrointestinal bleeding	10 (12.0)	4 (10.3)	6 (13.6)	0.893
Bowel obstruction	2 (2.4)	1 (2.6)	1 (2.3)	1.000
Hypoalbuminemia	45 (54.2)	21 (53.8)	24 (54.5)	0.949
Liver dysfunction	10 (12.0)	2 (5.1)	8 (18.2)	0.137
**Neuropsychiatric disease**				
Stroke	14 (16.9)	2 (5.1)	12 (27.3)	**0.007**
Others ^a^	3 (3.6)	0 (0)	3 (6.8)	0.284
**ENT involvement**	9 (10.8)	3 (7.7)	6 (13.6)	0.606
**Hematologic involvement**				
Leukopenia	4 (4.8)	2 (5.1)	2 (4.5)	1.000
Anemia	69 (83.1)	29 (74.4)	40 (90.9)	**0.044**
Thrombocytopenia	11 (13.3)	6 (15.4)	5 (11.4)	0.590
MAS	1 (1.2)	0 (0)	1 (2.3)	1.000
TTP	1 (1.2)	0 (0)	1 (2.3)	1.000
**Infection**				
Pneumonia	72 (86.7)	30 (76.9)	42 (95.5)	**0.013**
Urinary tract infection	3 (3.6)	0 (0)	3 (6.8)	0.284
Sepsis	9 (10.8)	2 (5.1)	7 (15.9)	0.221
**Shock**	27 (32.5)	2 (5.1)	25 (56.8)	**<0.001**

*Values highlighted in bold represent statistically significant P values (P < 0.05)*.

a *Other neuropsychiatric disease including one patient with seizure, one with uremic encephalopathy and one with lower-extremity numbness*.

*AAV, antineutrophil cytoplasmic antibody-associated vasculitis; ICU, intensive care unit; ENT, Ear, Nose, and Throat; MAS, macrophage activation syndrome; TTP, thrombotic thrombocytopenic purpura*.

Renal insufficiency occurred in 59 patients (71.1%) while 38 patients (45.8%) had glomerulonephritis (Table 3). However, there were no significant differences between non-survivors and survivors in terms of renal involvement. More non-survivors suffered from stroke (27.3% vs. 5.1%) and anemia (90.9% vs 74.4%) than survivors. Besides, shock was more frequent in non-survivors (56.8%) than survivors (5.1%). Pneumonia was reported in 72 patients (86.7%), with a significantly higher proportion in non-survivors (95.5% vs. 76.9%). The most common pathogens identified by culture of sputum or bronchoalveolar lavage fluid were *Acinetobacter baumannii* (12, 16.7%), followed by *Candida albicans* (10, 13.9%), *Klebsiella pneumonia* (8, 11.1%), *Aspergillus* (7, 9.7%), virus (7, 9.7%), *Pseudomonas aeruginosa* (5, 6.9%) and *Pneumocystis carinii* (5, 6.9%). However, 20 patients suffered from mixed infections while 30 were infected with unknown infectious agents. Urinary tract infection was detected in three patients (3.6%). Urine cultures of the patients revealed that they were separately infected with *Klebsiella pneumonia, Enterococcus faecalis* and *Candida tropicalis*. Sepsis was detected in nine patients (10.8%), with *Klebsiella pneumonia* (2, 22.2%) and *Staphylococcus aureus* (2, 22.2%) being the dominant causal bacteria.

The laboratory data collected during the ICU admission for non-survivors and survivors are presented in Supplementary Table 2. Non-survivors had lower level of hemoglobin (78.62 ± 21.83 g/L) than survivors (91.80 ± 27.94 g/L). Moreover, blood urea nitrogen, cardiac troponin I and procalcitonin were significantly higher for non-survivors than that for survivors. Most patients (75.9%) with AAV admitted to the ICU had positive p-ANCA while only 12.0% of the patients had positive c-ANCA. There was no statistical difference between non-survivors' and survivors' groups in the ANCA subtypes.

### Treatment Strategies for Non-survivors and Survivors

Strategies for management of the 83 AAV patients during admission in ICU are provided in Table 4. Thirty-two patients (72.7%) in non-survivors and 28 patients (71.8%) in survivors' group received glucocorticoids. Cyclophosphamide was administered intravenously to one patient in the survivors' group and orally to another patient in the non-survivors' group. Our results indicated that there were more patients from the non-survivors' group (56.8%) in need of catecholamines to maintain normal blood pressure than from the survivors' group (7.7%). In total, 43 patients (51.8%) required mechanical ventilation during the ICU, among whom 32 patients (38.6%) received endotracheal intubation. A significantly higher proportion of non-survivors needed mechanical ventilation and endotracheal intubation than survivors (*P* < 0.001). Plasma exchange was performed in 11 patients (13.3%) and hemodialysis was performed in 33 patients (39.8%), with no statistical difference between non-survivors' and survivors' groups.

**Table 4:** Treatment for 83 AAV patients in ICU.

**Treatment**	**Total** **(***n*** = 83)**	**Survivors** **(***n*** = 39)**	**Non-survivors** **(***n*** = 44)**	***P*** **-value**
Glucocorticoids	60 (72.3)	28 (71.8)	32 (72.7)	0.925
Pulsed methylprednisolone	12 (14.5)	3 (7.7)	9 (20.5)	0.099
Oral cyclophosphamide	1 (1.2)	0 (0)	1 (2.3)	1.000
Intravenous cyclophosphamide	1 (1.2)	1 (2.6)	0 (0)	0.952
IVIG	26 (31.3)	10 (25.6)	16 (36.4)	0.293
Catecholamines	28 (33.7)	3 (7.7)	25 (56.8)	**<0.001**
Antibiotics	80 (96.4)	38 (97.4)	42 (95.5)	1.000
Plasma exchange	11 (13.3)	4 (10.3)	7 (15.9)	0.448
Hemodialysis	33 (39.8)	15 (38.5)	18 (40.9)	0.820
Mechanical ventilation	43 (51.8)	8 (20.5)	35 (79.5)	**<0.001**
Endotracheal intubation	32 (38.6)	5 (12.8)	27 (61.4)	**<0.001**
Tracheotomy	3 (3.6)	1 (2.6)	2 (4.5)	1.000

*Values highlighted in bold represent statistically significant P values (P < 0.05)*.

*AAV, antineutrophil cytoplasmic antibody-associated vasculitis; ICU, intensive care unit; IVIG, intravenous immunoglobulin*.

### Mortality and Predictors of In-ICU Mortality for AAV Patients

In total 44 patients deceased in ICU, representing a mortality rate of 53%. The main cause of death was infection (27/44, 61.4%) followed by active vasculitis (15/44, 34.1%). The remaining two patients deceased of other reasons. One was suffering from cachexia due to advanced esophageal cancer while the other deceased of respiratory arrest due to cerebral hernia. When further analyzing the association between in-ICU death and respiratory failure, 36 patients (81.8%) died of respiratory failure with the causes of active vasculitis (13/36, 36.1%) or pulmonary infection (23/36, 63.9%). However, 18 patients (18/36, 50.0%) actually deceased of respiratory failure accompanied with renal failure or heart failure. Mortality rates among MPA (54.3%), GPA (45.5%) and EGPA (50%) were similar with no statistical difference. Mortality rates were comparable (*P* = 0.056) among different causes of ICU admission, including active vasculitis (52.5%), infection (54.3%) and other reasons (50.0%).

To identify the possible factors influencing the risk of in-ICU death for AAV patients, univariate and multivariate logistic regression analysis was performed (Table 5). Univariate logistic regression analysis found that APACHE II, BVAS, hemoglobin, pneumonia, pulmonary arterial hypertension, stroke, respiratory failure and shock were significantly associated with in-ICU death. The multivariate logistic regression analysis of the eight independent variables indicated that APACHE II (*OR* = 1.333, 95% *CI*: 1.031–1.722, *P* = 0.028) and respiratory failure (*OR* = 620.452, 95% *CI*: 11.495–33490.306, *P* = 0.002) were associated with in-ICU mortality. However, hemoglobin (*OR* = 0.919, 95% *CI*: 0.849–0.995, *P* = 0.037) was adversely associated with in-ICU mortality of AAV patients. The cut-off value of APACHE II was 14.5 determined by the ROC curve, with sensitivity of 79.5% and specificity of 79.5%. Besides, APACHE II more than 14.5 was significantly associated with in-ICU mortality (*OR* = 12.963, 95% *CI*: 4.560–36.849, *P* < 0.001).

**Table 5:** Risk factors of in-ICU mortality for AAV patients.

	**Univariate logistic analysis**		**Multivariate logistic analysis**	
	***OR*** **(95% ***CI***)**	***P*** **-value**	***OR*** **(95% ***CI***)**	***P*** **-value**
APACHE II	1.311 (1.161–1.479)	**<0.001**	1.333 (1.031–1.722)	**0.028**
BVAS	1.129 (1.061–1.202)	**<0.001**	0.823 (0.663–1.020)	0.076
Hemoglobin	0.979 (0.961–0.997)	**0.023**	0.919 (0.849–0.995)	**0.037**
AST	1.010 (0.993–1.027)	0.257	-	-
TBil	1.060 (0.984–1.142)	0.122	-	-
BUN	1.018 (0.989–1.047)	0.238	-	-
CNI	8.931 (0.386–206.485)	0.172	-	-
PCT	1.013 (0.976–1.051)	0.502	-	-
BGA-pH	0.041 (0.001–2.221)	0.117	-	-
Anemia	3.448 (0.984–12.086)	0.053	-	-
Pneumonia	6.300 (1.269–31.273)	**0.024**	241.791 (0.393–148,792.713)	0.094
Renal insufficiency	2.431 (0.916–6.451)	0.075	-	-
Glomerulonephritis	2.143 (0.886–5.183)	0.091	-	-
PAH	14.250 (1.756–115.613)	**0.013**	7.486 (0.128-436.908)	0.332
Stroke	6.937 (1.443–33.344)	**0.016**	18.511 (0.349–980.626)	0.150
Respiratory failure	70.000 (14.102–347.479)	**<0.001**	620.452 (11.495–33490.306)	**0.002**
Shock	24.342 (5.204–113.870)	**<0.001**	22.365 (0.965–518.384)	0.053

*Values highlighted in bold represent statistically significant P values (P < 0.05)*.

*AAV, antineutrophil cytoplasmic antibody-associated vasculitis; ICU, intensive care unit; APACHE II, Acute Physiology and Chronic Health Evaluation II; BVAS, Birmingham vasculitis active scoring system; AST: aspartate aminotransferase; TBil: total bilirubin; BUN, blood urea nitrogen; CNI, cardiac troponin I; PCT, procalcitonin; BGA-pH, blood gas analysis-power of hydrogen; PAH, pulmonary arterial hypertension*.

### Characteristics of AAV Patients With Diffuse Alveolar Hemorrhage

As one of common causes for ICU admission and respiratory failure, further analysis of patients with DAH was presented (Table 6). In total, 19 patients (22.9%) suffered from DAH in our study, among whom 12 (63.2%) died in the ICU. Eleven patients (57.9%) were female, with a mean age of 63 ± 13 years of all the patients. All of the patients complicated with DAH were ANCA positive (p/MPO-ANCA positive: 73.7%, and c/PR3-ANCA positive: 26.3%). The groups between non-survivors and survivors were similar regarding gender, age, classification of AAV and ANCA types. Most of the patients (78.9%) were newly diagnosed with AAV, with DAH occurring as the first manifestation. Hemoptysis was presented in 47.4% and cough was presented in 52.6%. Fourteen patients (73.7%) suffered from respiratory failure and 14 (73.7%) were accompanied with renal insufficiency. More non-survivors suffered from respiratory failure (91.7% vs. 42.9%) and shock (50.0% vs. 0%) than survivors. The median of APACHE II score at ICU admission was higher in non-survivors' group [16 (14–26)] than that in survivors' group [10 (9–14)], whereas the median of BVAS scores were comparable between the two groups.

**Table 6:** Characteristics of 19 AAV patients with diffuse alveolar hemorrhage.

**Characteristics**	**Total** **(***n*** = 19)**	**Survivors** **(***n*** = 7)**	**Non-survivors** **(***n*** = 12)**	***P*** **-value**
**Demographics**				
Age (years, mean ± SD)	63 ± 13	61 ± 13	64 ± 14	0.702
Female gender (n, %)	11 (57.9)	3 (42.9)	8 (66.7)	0.377
**Classification of AAV (** ***n*** **, %)**				0.305
MPA	14 (73.7)	4 (57.1)	10 (83.3)	
GPA	5 (26.3)	3 (42.9)	2 (16.7)	
EGPA	0 (0)	-	-	
**Autoantibodies (** ***n*** **, %)**				0.305
c/PR3-ANCA	5 (26.3)	3 (42.9)	2 (16.7)	
p/MPO-ANCA	14 (73.7)	4 (57.1)	10 (83.3)	
**Newly diagnosed as AAV (** ***n*** **, %)**	15 (78.9)	5 (71.4)	10 (83.3)	0.603
**Length of stay in ICU (day), M (Q1-Q3)**	6 (2–11)	6 (2–11)	6 (2-8)	0.670
**Clinical manifestations (** ***n*** **, %)**				
Hemoptysis	9 (47.4)	4 (57.1)	5 (41.7)	0.650
Cough	10 (52.6)	3 (42.9)	7 (58.3)	0.650
Respiratory failure	14 (73.7)	3 (42.9)	11 (91.7)	**0.038**
Renal insufficiency	14 (73.7)	4 (57.1)	10 (83.3)	0.305
Heart failure	15 (78.9)	5 (71.4)	10 (83.3)	0.603
Shock	6 (31.6)	0 (0)	6 (50.0)	**0.044**
Anemia	18 (94.7)	6 (85.7)	12 (100)	0.368
Infection	18 (94.7)	6 (85.7)	12 (100)	0.368
**Disease and severity assessment scores**				
APACHE II, M (Q1–Q3)	15 (11–18)	10 (9–14)	16 (14-26)	**0.008**
BVAS, M (Q1–Q3)	24 (21-30)	20 (15-26)	25 (23-32)	0.127
**In-ICU management**				
Mechanical ventilation	11 (57.9)	2 (28.6)	9 (75.0)	0.074
Endotracheal intubation	7 (36.8)	1 (14.3)	6 (50.0)	0.173
Plasma exchange	4 (21.1)	2 (28.6)	2 (16.7)	0.603
Hemodialysis	8 (42.1)	3 (42.9)	5 (41.7)	1.000
Catecholamines	7 (36.8)	0 (0)	7 (58.3)	**0.017**
Glucocorticoids	14 (73.7)	6 (85.7)	8 (66.7)	0.603
Pulsed methylprednisolone	6 (31.6)	2 (28.6)	4 (33.3)	1.000
Cyclophosphamide	0 (0)	-	-	-
IVIG	6 (31.6)	4 (47.1)	2 (16.7)	0.129

*Values highlighted in bold represent statistically significant P values (P < 0.05)*.

*AAV, antineutrophil cytoplasmic antibody-associated vasculitis; ICU, intensive care unit; MPA, microscopic polyangiitis; GPA, granulomatosis with polyangiitis; EGPA, eosinophilic granulomatosis with polyangiitis; c/PR3-ANCA, cytoplasmic or proteinase-3 antineutrophil cytoplasmic antibody; p/MPO-ANCA: perinuclear or myeloperoxidase antineutrophil cytoplasmic antibody; APACHE II, Acute Physiology and Chronic Health Evaluation II; BVAS, Birmingham vasculitis active scoring system, IVIG, intravenous immunoglobulin*.

Among 11 patients (57.9%) who required mechanical ventilation, endotracheal intubation was performed in 7 (36.8%). Four patients (21.1%) were treated with plasma exchange and 8 (42.1%) with hemodialysis, while no statistical difference was detected between non-survivors' and survivors' groups. A significantly higher proportion of non-survivors (58.3%) needed catecholamines than survivors (0%). Fourteen patients (73.7%) were administered with glucocorticoids, among whom 6 (31.6%) received pulsed methylprednisolone. All of the 12 non-survivors deceased of respiratory failure, mainly due to active vasculitis or infection complications. Among the remaining seven survivors, one patient suffered from DAH relapse 2 months after admission to the ICU.

### Establishment of a Nomogram for Predicting the Risk of In-ICU Mortality for AAV Patients

**Figure 2:**
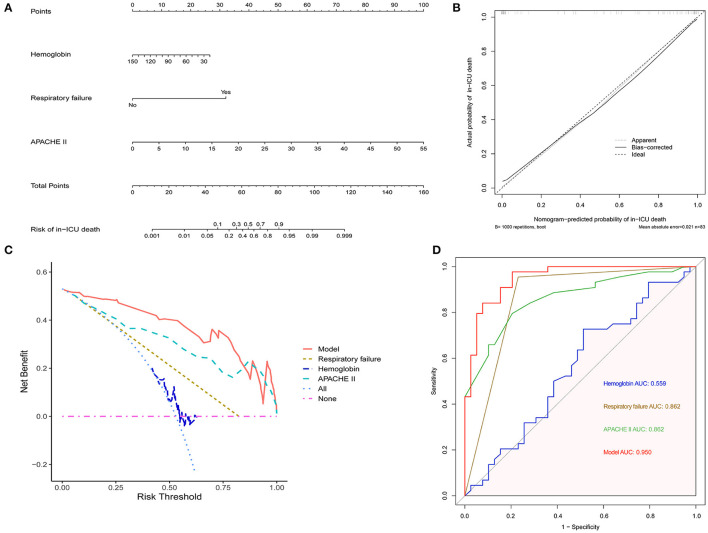
The establishment of nomogram to predict in-ICU mortality of AAV patients. **(A)** The nomogram for predicting in-ICU mortality of AAV patients. **(B)** Calibration curve of the nomogram. **(C)** DCA of the nomogram. **(D)** ROC curves comparing the nomogram with APACHE II, hemoglobin and respiratory failure. AAV, antineutrophil cytoplasmic antibody-associated vasculitis; ICU, intensive care unit; APACHE II, Acute Physiology and Chronic Health Evaluation II; DCA, decision curve analysis; ROC, receiver operating characteristic; AUC, area under curve.

To further evaluate the in-ICU death risk of AAV patients, a nomogram was developed based on independent factors of in-ICU mortality by multivariate logistic regression analysis (Figure 2A). The calibration curve of the established nomogram for the prediction of in-ICU death risk of AAV patients demonstrated good agreement in this cohort (Figure 2B). The results of DCA showed that the established nomogram had a good net benefit and a wide range of threshold probability (Figure 2C). The comparison of different ROC indicated that the established nomogram had a better predictive value (Figure 2D). Therefore, the nomogram we established has a significant clinical use.

### Long-Term Outcome of AAV Patients

**Figure 3:**
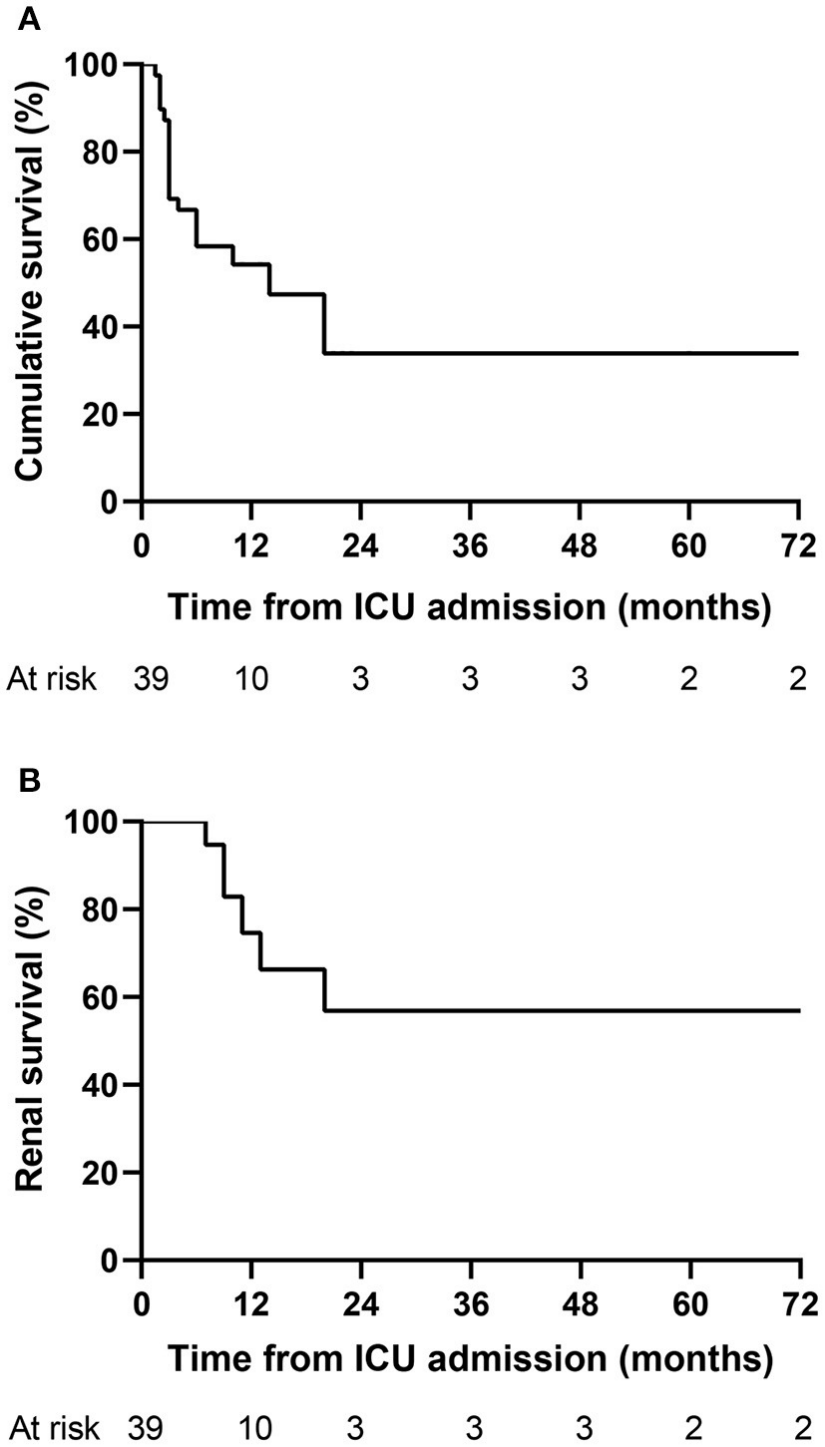
Kaplan–Meier curves for the long-term outcome of AAV patients. **(A)** Cumulative survival of 39 patients that survived to the first ICU stay. **(B)** Renal survival of 39 patients that survived to the first ICU stay. AAV, antineutrophil cytoplasmic antibody-associated vasculitis; ICU, intensive care unit.

Given the relatively high in-ICU mortality in our study, we further explored the long-term outcome of 39 patients that survived to the first ICU stay (Figure 3). Twelve patients (30.8%) deceased within 3 months after admission to the ICU. Kaplan–Meier analysis showed that the cumulative survival rates of 39 patients at 0.5, 1, and 5 years were 58.3%, 54.2%, and 33.9%, respectively (Figure 3A), and the median survival time was 14 months. The renal survival rates at 0.5, 1, and 5 years were 100%, 74.6%, and 56.8%, respectively (Figure 3B). During the follow-up period, 20 patients died, with the overall mortality of 51.3%. ESRD occurred in seven patients (36.8%) among the remaining 19 survivors, with one patients survived with a transplanted kidney and the others received long-term renal-replacement therapy.

## Discussion

AAV is one of common systemic rheumatic diseases (SRD) requiring ICU admission (9, 32) and represents a challenge in the ICU. Despite the improvement of therapeutic strategies, the in-ICU mortality of AAV is still high, ranging from 15.5% to 58.8% (8, 11, 12). Several studies have focused on the outcome of AAV patients in the ICU (6, 8, 11, 17, 33). However, most of them are small-sized and data for the Asian populations are lacking. Here, we conducted a single-center, retrospective study in a tertiary medical institution of China, to analyze the clinical features of AAV patients admitted to the ICU and explore the risk factors of in-ICU mortality. A nomogram was established using the identified risk factors to predict the risk of in-ICU mortality of AAV patients.

In this study, active vasculitis was identified as the leading cause of ICU admission. Active vasculitis mainly included diseases such as acute renal insufficiency, diffuse alveolar hemorrhage and pulmonary-renal syndrome. A multicenter retrospective study showed that the main reason of AAV patients admitted to the ICU was respiratory failure due to massive hemoptysis (11). Frausova et al. (6) illustrated in their study that the most frequent cause of ICU admission was active vasculitis with pulmonary-renal syndrome. The manifestations of AAV are various, including non-specific clinical symptoms like weight loss, malaise, arthralgia and myalgia (2), while some patients may manifest with severe or life-threatening diseases thereby requiring ICU admission. Therefore, AAV should be considered as a possible diagnosis if a patient admitted to the ICU has unexplained severe systemic manifestations, mainly pulmonary or renal failure.

Infection was reported in the majority of our patients during the ICU, with pneumonia being the most common diagnosis. Since AAV patients are usually treated with high doses of glucocorticoids accompanied with immunosuppressive therapies, opportunistic infections, such as *Pneumocystis carinii, Aspergillus* and *Cytomegalovirus* are frequently observed (7). In addition, invasive operations like endotracheal intubation, central venous catheterization may lead to nosocomial infections (6, 34). In this study, the most common infectious pathogens were *Acinetobacter baumannii, Candida albicans, Klebsiella pneumonia* and *Aspergillus*, mainly belonging to Gram-negative bacteria and Fungi. Some studies illustrated that Gram-negative bacteria were the most frequent pathogens (8, 11). Other studies did not show a significant predominance of pathogens (34, 35) probably due to small sample size and insufficient data. Therefore, there is a need to test for more infectious pathogens in AAV patients admitted the ICU.

For AAV patients, the most commonly and severely affected systems are the respiratory and renal systems. Main presentations of pulmonary involvement may be various among different AAV categories (36–38). The study of Hruskova et al. demonstrated that severe DAH was more frequent in c/PR3-ANCA than p/MPO-ANCA-positive patients (67.9% vs. 32.1%) (39). The prevalence of DAH was comparable between the groups of PR3-AAV and MPO-AAV in the Rituximab in ANCA-associated Vasculitis (RAVE) trial (40). Although 73.7% of patients with DAH was p/MPO-ANCA-positive in our study, more c/PR3-ANCA-positive patients suffered from DAH when we further analyzed in different types of ANCAs. However, some studies reported that DAH was more frequent in MPA or p/MPO-ANCA-positive patients compared to GPA (37, 41). ILD was reported to be more common in MPA or p/MPO-ANCA-positive patients (42, 43). Our study revealed a higher prevalence of ILD in p/MPO-ANCA-positive patients, though failing to present a statistical significance. However, more researches regarding heterogeneity of pulmonary presentations are needed in the future. In our study, the majority of patients suffered from renal insufficiency. However, no significant difference was found between in-ICU non-survivors and survivors in renal insufficiency possibly because of timely renal replacement therapy performed for most of the patients during the ICU. Previous studies demonstrated that renal involvement was associated with worse long-term prognosis of AAV patients (41, 44), highlighting the importance of earlier diagnosis and prompt therapy.

The in-ICU mortality of our study group was 53%, which was comparable with the study results of Biscetti et al. (17) and Befort et al. (34). However, other studies have reported results of ICU mortality rates that are inconsistent with ours (6, 11, 45). The major reasons may be the heterogeneity of the included population and different follow-up durations. Similar to the results of our study, infection was the most frequent reason of in-ICU death in previous studies (6, 34). Besides, ICU mortality was higher in AAV patients admitted for infectious complication than for exacerbation of rheumatic diseases (46), though which was not significant in our study. As reported by Flossmann et al., infection was one of main causes of death within (48%) and after (20%) the first year (5). Therefore, it is necessary for clinicians to test sputum, blood, urine and catheters in order to identify and treat infections timely.

Identification of the predictors of in-ICU mortality of AAV patients is vital. We identified eight factors related to in-ICU mortality by univariate logistic analysis. However, when incorporating all of them in the multivariate logistic analysis, only APACHE II and respiratory failure were associated with an increased risk of in-ICU mortality while hemoglobin was an independent protective factor. Today, administration with glucocorticoids accompanied with cyclophosphamide or rituximab is still highly recommended for remission induction in AAV patients (22). However, immunosuppressive drugs are not routinely prescribed for severe vasculitis in ICU, possibly due to less awareness of the underlying disease or the worry of severe side effects concerning the immunosuppressant, including infections, bone marrow suppression and hemorrhagic cystitis (47, 48). In our study, only two patients received cyclophosphamide during ICU admission. Most of the patients suffered from infection at the moment of or during ICU admission, so physicians in ICU always considered prudently and were hesitant to prescribe immunosuppressive agents, such as cyclophosphamide. Mechanical ventilation, endotracheal intubation and administration of catecholamine were considered as necessary therapeutic measures in this study. Therefore, these therapies were not included in logistic regression analysis. The APACHE II is a simple and accurate assessment system of the severity of disease in critically ill patients (14). Several studies reported a higher APACHE II score for non-survivors of AAV patients (6, 11, 35). This indicated that APACHE II could be used to predict the outcome of AAV patients admitted to the ICU.

Respiratory failure, probably resulting from diffuse alveolar hemorrhage or pulmonary fibrosis accompanied with severe pneumonia, was another independent risk factor of in-ICU death. In a study that focused on the outcome of rheumatology patients with acute respiratory failure, the in-ICU mortality rate was high (59.8%), and vasculitis was associated with increased mortality (49). Ozdemir et al. showed in their study that 5 (25.0%) patients died of respiratory failure, with massive hemoptysis (four patients) and resistant pulmonary edema (one patient) (11). Demiselle et al. reported that in-ICU death of five patients (33.0%) was attributed to multiple organ failure likely due to sepsis (12). As respiratory failure was one of independent risk factors in our study, it was also a vital cause of in-ICU death among the non-survivors. Patients with DAH had a higher mortality (63.2%) compared with the overall mortality (53.0%) of all the patients in our study. All of them deceased of respiratory failure, further highlighting the importance of vigilance and prompt therapy to it. Arterial blood gas analysis is a convenient and direct way to monitor the saturation and pressure of oxygen. It can also be used to compute the oxygenation index (PaO_2_/FiO_2_). So arterial blood gas analysis may be helpful for clinicians to monitor if the AAV patients are suffering from respiratory failure and try to remove the pathogenic factors in time.

In this study, hemoglobin was an independent protective factor from ICU mortality. Most of the patients in our cohort suffered from anemia. Chronic anemia may result from renal involvement. Additionally, a higher proportion of non-survivors had diffuse alveolar hemorrhage and sepsis which might lead to reduced hemoglobin in a short duration. Ge et al. illustrated that a lower hemoglobin level (<90 g/L) was significantly associated with a greater risk for poor renal survival in patients with MPO-ANCA-associated glomerulonephritis (50). Flossmann et al. revealed that lower hemoglobin was one of significant negative prognostic factors for AAV patients' long-term survival (5). However, whether hemoglobin is associated with higher in-ICU mortality need to be further studied. The BVAS comprises ten systems and is used to assess disease activity of AAV (21). In this study, BVAS was not associated with mortality risk in multivariate analysis. However, Biscetti et al. (17) found that BVAS>10 in ICU might be a practical tool to predict in-ICU mortality. Befort et al. (34) reported that BVAS score (*OR* = 1.16, 95% *CI*: 1.01–1.34) was predictive of mortality in the ICU. However, more research is needed to establish whether BVAS can be used to predict the outcome of AAV patients in the ICU.

This study has several limitations which can form the basis for further research. Firstly, this is a single-center, retrospective study. Therefore, information bias maybe exists. As a consequence, our results as well as the established nomogram need to be interpreted cautiously. Secondly, due to the relatively high in-ICU mortality in our study, a relatively small number of patients were remained during the follow-up. Therefore, predictive factors of long-term prognosis for critically ill AAV patients should be further explored in a larger cohort. The results in our study should be confirmed by multi-center and prospective clinical research studies in the future.

## Conclusion

In this study, active vasculitis was identified as the most common reason for ICU admission. The in-ICU mortality of AAV patients was 53%. The main cause of in-ICU death was infection. APACHE II and respiratory failure were independent risk factors while hemoglobin was an independent protective factor of in-ICU deaths for AAV patients admitted to the ICU. Finally, a simple model for predicting in-ICU mortality of AAV patients was established for clinical application.

## Data Availability Statement

The original contributions presented in the study are included in the article/Supplementary Material, further inquiries can be directed to the corresponding author.

## Ethics Statement

The studies involving human participants were reviewed and approved by Ethical Committee of the First Affiliated Hospital of Zhengzhou University. The Ethics Committee waived the requirement of written informed consent for participation.

## Author Contributions

SL, YZ, and JG conceived and participated in the design of the study. YZ, PZ, XD, and LZ collected the clinical data of patients. YZ, PZ, XS, and NG analyzed the data. SL, LZ, and XS performed the conceptualization. The manuscript was written by YZ and JG. All authors reviewed and approved the final manuscript.

## Funding

This work was supported by Henan Province Medical Science and Technology Projects [SB201901020] and Henan Province Medical Science and Technology Program Provincial and Ministerial Joint Projects [SBGJ202003024].

## Conflict of Interest

The authors declare that the research was conducted in the absence of any commercial or financial relationships that could be construed as a potential conflict of interest.

## Publisher's Note

All claims expressed in this article are solely those of the authors and do not necessarily represent those of their affiliated organizations, or those of the publisher, the editors and the reviewers. Any product that may be evaluated in this article, or claim that may be made by its manufacturer, is not guaranteed or endorsed by the publisher.

## Abbreviations

AAV: antineutrophil cytoplasmic antibody-associated vasculitis
ICU: intensive care unit
MPA: microscopic polyangiitis
GPA: granulomatosis with polyangiitis
EGPA: eosinophilic granulomatosis with polyangiitis
APACHE II: Acute Physiology and Chronic Health Evaluation II
SOFA: Sequential Organ Failure Assessment
SAPS II: Simplified Acute Physiologic Score II
BVAS: Birmingham vasculitis activity score system
ESRD: end-stage renal disease
GFR: glomerular filtration rate
DAH: diffuse alveolar hemorrhage
ILD: interstitial lung disease
ENT, ear: nose and throat
CRP: C-reactive protein
ESR: erythrocyte sedimentation rate
SRD: systemic rheumatoid disease
DCA: decision curve analysis
ROC: receiver operating characteristic.
