# Supplementary material for: Predictors of Mortality in Critically Ill Patients With Antineutrophil Cytoplasmic Antibody-Associated Vasculitis
Source: Front Med (Lausanne). 2021 Oct 25;8:762004. doi: 10.3389/fmed.2021.762004 (PMC8573203; doi:10.3389/fmed.2021.762004)
Supplement: Supplementary file 1 [file Table_1.DOCX]

**Supplementary Material Table S1** Manifestations of pulmonary involvement in 73 ANCA-positive patients

| Manifestations (n, %) | p/MPO-ANCA positive  (n=63) | c/PR3-ANCA positive  (n=10) | *P*-value |
| --- | --- | --- | --- |
| Cough | 24 (38.1) | 5 (50.0) | 0.505 |
| Hemoptysis | 9 (14.3) | 2 (20.0) | 1.000 |
| Diffuse alveolar hemorrhage | 14 (22.2) | 5 (50.0) | 0.114 |
| Interstitial lung disease | 20 (31.7) | 1 (10.0) | 0.263 |
| Pulmonary arterial hypertension | 10 (15.9) | 1 (10.0) | 0.701 |
| Pulmonary nodules | 7 (11.1) | 1 (10.0) | 1.000 |
| Pulmonary infiltrates | 57 (90.5) | 10 (100.0) | 0.587 |
| Pleural effusion | 38 (60.3) | 6 (60.0) | 1.000 |
| Pulmonary embolism | 3 (4.8) | 0 (0) | 1.000 |
| Respiratory failure | 38 (60.3) | 7 (70.0) | 0.732 |

Abbreviation: ANCA, antineutrophil cytoplasmic antibody; p/MPO-ANCA: perinuclear or myeloperoxidase antineutrophil cytoplasmic antibody; c/PR3-ANCA, cytoplasmic or proteinase-3 antineutrophil cytoplasmic antibody.
