# Supplementary material for: Predictors of Mortality in Critically Ill Patients With Antineutrophil Cytoplasmic Antibody-Associated Vasculitis
Source: Front Med (Lausanne). 2021 Oct 25;8:762004. doi: 10.3389/fmed.2021.762004 (PMC8573203; doi:10.3389/fmed.2021.762004)
Supplement: Supplementary file 2 [file Table_2.DOCX]

**Supplementary Material Table S2** Some laboratory data of 83 AAV patients

|  | Total  (n=83) | Survivors  (n=39) | Non-survivors (n=44) | *P* -value |
| --- | --- | --- | --- | --- |
| WBC (×10^9^), M (Q1-Q3) | 10.26 (7.10, 15.62) | 8.79 (7.20, 14.30) | 10.42 (6.66, 17.97) | 0.414 |
| Hemoglobin (g/L, mean ± SD) | 84.81 ± 25.60 | 91.80 ± 27.94 | 78.62 ± 21.83 | **0.020** |
| Platelet (×10^9^), M (Q1-Q3) | 179.00 (133.00, 249.00) | 179.00 (153.00, 319.00) | 165.50 (122.50, 234.50) | 0.250 |
| Neutrophils (×10^9^), M (Q1-Q3) | 8.52 (6.03, 13.75) | 7.17 (5.52, 12.42) | 9.83 (6.20, 14.74) | 0.195 |
| Lymphocytes (×10^9^), M (Q1-Q3) | 0.73 (0.33, 1.20) | 0.79 (0.36, 1.39) | 0.71 (0.32, 1.06) | 0.491 |
| Albumin (g/L, mean ± SD) | 29.41 ± 4.93 | 29.57 ± 4.45 | 29.26 ±5.37 | 0.773 |
| Globin (g/L, mean ± SD) | 31.97 ± 6.36 | 31.64 ±5.67 | 32.26 ±6.97 | 0.657 |
| ALT (U/L), M (Q1-Q3) | 19.00 (10.00, 35.00) | 16.00 (10.00, 27.00) | 21.00 (9.25, 43.50) | 0.283 |
| AST (U/L), M (Q1-Q3) | 22.00 (18.00, 42.00) | 21.00 (18.00, 33.00) | 25.00 (18.00, 33.00) | 0.064 |
| TBil (mmol/L), M (Q1-Q3) | 9.00 (7.00, 12.90) | 8.00 (6.90, 11,30) | 10.00 (7.40, 11.38) | 0.089 |
| DBil (mmol/L), M (Q1-Q3) | 3.20 (1.20, 4.80) | 2.90 (1.20, 4.59) | 3.45 (1.20, 7.05) | 0.363 |
| Urine protein (n, %) | 63 (75.9) | 27 (69.2) | 36 (81.8) | 0.181 |
| Scr (μmol/L), M (Q1-Q3) | 242.20 (77.40, 573.20) | 242.20 (63.00, 580.00) | 262.60 (105.75, 535.33) | 0.538 |
| GFR (ml/min/1.73m^2^), M (Q1-Q3) | 18.06 (6.70, 79.44) | 30.56 (9.77, 95.70) | 17.24 (8.19, 44.57) | 0.168 |
| BUN (mmol/L), M (Q1-Q3) | 18.39 (9.84, 28.20) | 14.60 (5.80, 24.20) | 21.05 (10.95, 29.65) | **0.047** |
| BNP (pg/ml), M (Q1-Q3) | 6818.50 (1184.50, 27863.75) | 4410.00 (619.69, 35000.00) | 7792.87 (3069.00, 26985.00) | 0.248 |
| CNI (μg/L), M (Q1-Q3) | 0.048 (0.022, 0.115) | 0.026 (0.015, 0.087) | 0.051 (0.028, 0.180) | **0.022** |
| ESR (mm/h), M (Q1-Q3) | 73.00 (32.50, 97.50) | 73.00 (42.00, 102.50) | 72.50 (30.25, 97.00) | 0.718 |
| CRP (mg/L, mean ± SD) | 113.38 ± 75.58 | 109.25 ± 77.56 | 117.17 ± 74.57 | 0.662 |
| PCT (ng/L), M (Q1-Q3) | 0.57 (0.22, 2.04) | 0.42 (0.11, 1.85) | 0.87 (0.31, 2.29) | **0.044** |
| BGA-pH, M (Q1-Q3) | 7.43 (7.32, 7.48) | 7.45 (7.37, 7.50) | 7.42 (7.31, 7.46) | 0.092 |
| c-ANCA (n, %) | 10 (12.0) | 5 (12.8) | 5 (11.4) | 1.000 |
| p-ANCA (n, %) | 63 (75.9) | 29 (74.4) | 34 (77.3) | 0.757 |
| PR3-ANCA (IU/ml), M (Q1-Q3) | 4.25 (2.00, 10.58) | 3.70 (2.00, 8.50) | 5.50 (2.00, 11.68) | 0.536 |
| MPO-ANCA (IU/ml), M (Q1-Q3) | 78.20 (9.78, 213.80) | 90.50 (10.90, 254.00) | 76.40 (9.00, 170.28) | 0.541 |

Values highlighted in bold represent statistically significant *P* values (*P* < 0.05). Abbreviations: AAV, antineutrophil cytoplasmic antibody-associated vasculitis; ALT: alanine aminotransferase; AST: aspartate aminotransferase; TBil: total bilirubin; DBil: direct bilirubin; Scr, serum creatinine; GFR, glomerular filtration rate; BUN, blood urea nitrogen; BNP, Brain natriuretic peptide; CNI, cardiac troponin I; ESR, erythrocyte sedimentation rate; CRP, C-reactive protein; PCT, procalcitonin; BGA-pH, blood gas analysis - power of hydrogen; c-ANCA, cytoplasmic antineutrophil cytoplasmic antibody; p-ANCA: perinuclear antineutrophil cytoplasmic antibody; PR3-ANCA, proteinase-3 antineutrophil cytoplasmic antibody; MPO-ANCA, myeloperoxidase antineutrophil cytoplasmic antibody.
